# 1202. Impact of CTX-M Rapid Diagnostic Testing via Biofire® FilmArray® Blood Culture Identification 2 Panel on Early Optimization of Antibiotic Treatment for *Enterobacterales* Bacteremia

**DOI:** 10.1093/ofid/ofad500.1042

**Published:** 2023-11-27

**Authors:** Brian Kim, Kevin Kurator, Oscar E Gallardo Huizar, Niki Arab, Arthur Jeng

**Affiliations:** Olive View-UCLA Medical Center, Sylmar, California; Olive View-UCLA Medical Center, Sylmar, California; Olive View-UCLA, Los Angeles, California; Olive View- UCLA Medical Center, Los Angeles, California; Olive View UCLA Medical Center/UCLA School of Medicine, Sylmar, California

## Abstract

**Background:**

Biofire® FilmArray® Blood Culture Identification 2 Panel (BCID2), compared to the original version, expands microbial targets and tests for seven additional resistance genes, including detection of CTX-M Extended-Spectrum Beta-Lactamase (ESBL). At Olive View-UCLA Medical Center (Sylmar, CA), BCID2 supplanted the original version for antimicrobial stewardship rapid diagnostic testing (RDT) of blood cultures (Bcx). We reviewed the impact of BCID2 CTX-M RDT on early optimization of antibiotic (abx) treatment (tx) for *Enterobacterales* bacteremia.

**Methods:**

Retrospective chart review of adults (≥ 18 years) admitted with BCID2 detection of *Enterobacterales* from April 1, 2022 to March 31, 2023. Exclusion criteria included detection of carbapenemase, concomitant infection warranting continuation of empiric abx despite BCID2 result, suspected contamination of Bcx, and BCID2 detection of *Serratia*, *Enterobacter*, or *Enterobacterales* without identification of species. ESBL-active abx (EAA) was defined as either amikacin or a carbapenem; non-EAA was any abx except amikacin or a carbapenem. Days of therapy (DOT) saved was calculated by the difference in days between abx change and Bcx finalization (CF).

**Results:**

120 BCID2 results met study inclusion. CTX-M was detected (CTX-Mpos) in 44 (37%) and was not detected (CTX-Mneg) in 76 (63%). In CTX-Mpos, 29 (66%) were started on an empiric non-EAA, of which 28 (97%) resulted in a change to an EAA < 24 hours (H) of BCID2 result; all 29 had a change to an EAA prior to CF. An average of 3 DOT [IQR 2-5] was saved. Five had a history (hx) of ESBL from any source. In CTX-Mneg, 13 (17%) were started on an empiric EAA, of which 8 (62%) had a change to a non-EAA < 24 H of BCID2 result. One had a change to a non-EAA ≥ 24 H of BCID2 result but prior to CF. Four had a change to a non-EAA after CF. An average of 3 DOT [IQR 2-3] was saved. Seven had a hx of ESBL from any source. BCID2 CTX-M results were 100% congruent with the presence or absence of ESBL on Bcx, respectively.

**Conclusion:**

BCID2 CTX-M RDT resulted in early optimization of abx tx in the majority of cases, leading to early escalation of non-EAA empiric tx against ESBL-pathogens and early de-escalation of EAA against non-ESBL pathogens. Rapid diagnostic testing for CTX-M is a valuable tool for antimicrobial stewardship.

**Disclosures:**

**All Authors**: No reported disclosures

